# Beneficial Effects of Paeoniflorin Enriched Extract on Blood Pressure Variability and Target Organ Damage in Spontaneously Hypertensive Rats

**DOI:** 10.1155/2017/5816960

**Published:** 2017-01-24

**Authors:** Bo Li, Zheng-Biao Yang, Shan-Shan Lei, Jie Su, Min-Xia Pang, Chao Yin, Guo-Yang Chen, Chao-Wen Shan, Bo Chen, Hui-Ming Hu, Su-Hong Chen, Gui-Yuan Lv

**Affiliations:** ^1^Zhejiang Chinese Medical University, Hangzhou, Zhejiang 310053, China; ^2^Zhejiang University of Technology, Hangzhou, Zhejiang 310014, China; ^3^Zhejiang Academy of Medical Sciences, Hangzhou 310053, China; ^4^Wenzhou Medical University, Wenzhou, Zhejiang 325035, China

## Abstract

Blood pressure variability (BPV) is associated with the development and progression of severe target organ damage (TOD). This study aims to evaluate the protective effect of paeoniflorin enriched extract from Radix Paeoniae Alba (PG) on BPV and TOD in spontaneously hypertensive rats (SHR). All SHR were orally treated with distilled water, metoprolol (MP, 20 mg/kg), and PG (PG-H, 90 mg/kg or PG-L, 30 mg/kg) for a single time or daily for 7 weeks. The 24-hour dynamic blood pressure was monitored and then calculated BPV including long- and short-term systolic blood pressure variability (SBPV), diastolic blood pressure variability (DBPV), mean blood pressure variability (MBPV), and heart rate variability (HRV) as well as the 24-hour-SBP, 24-hour-DBP, and 24-hour-MBP. The protective effects of PG on TOD were observed by histopathologic and biochemical detection. The results indicated that long- and short-term SBPV, DBPV, MBPV, and HRV as well as 24-hour-SBP, 24-hour-DBP, and 24-hour-MBP showed no significant changes after single-dose administration of PG and significantly decreased after administration with PG for 7 weeks. PG could also markedly improve the damage of aorta, heart, kidney, and brain. This study suggested that PG could notably reduce BPV, stabilize blood pressure, and mitigate TOD in SHR.

## 1. Introduction

Hypertension is one of the most popular risk factors affecting cardiovascular disease. Blood pressure (BP) sustaining high systolic pressure ≥140 mmHg or diastolic pressure ≥90 mmHg may be defined as hypertension [1]. According to epidemiology research, the occurrence of hypertension is persistent and shows an increasing trend. The global population suffering from hypertension is predicted to reach 1.56 billion by 2025 [2]. About 25.2% of people over 18 years old were diagnosed with hypertension in 2012 in China and almost 35.5% in Beijing [3]. The problem is shown not only by the large number of illness, but also by severe incidental symptoms of target organ damage (TOD). Fortunately, studies have been conducted on blood pressure variability (BPV) as an important risk factor inducing TOD [4, 5].

BPV is associated with the development and progression of severe target organ lesion of the brain, heart, kidney, and vessels, together with increased risk of cardiovascular disease and increased mortality. Thus, the treatment of hypertension should aim not only at decreasing BP but also at reducing BPV [6]. In recent decades, drug treatment of hypertension has focused on controlling BPV. Visit-to-visit research had found that antihypertensive therapeutic drugs of calcium channel blockers (CCB)/diuretics could reduce variability in systolic blood pressure (SBP) and angiotensin-receptor blocker (ARB)/CCB could reduce the long-term variability [7].

Radix Paeoniae Alba (RPA), with paeoniflorin as principal bioactive component, is a traditional Chinese medicine that lowers blood pressure and exhibits anti-inflammatory and antioxidative properties, among others [8]. An increasing number of studies found that paeoniflorin or extract of RPA exerts a positive effect on cardiovascular function [9–11]. The literature reported that the methanol extract of RPA and paeoniflorin could activate nitric oxide synthase (NOS) pathway, thereby increasing nitric oxide (NO) and NOS levels, as well as relaxing blood vessels in vitro [12]. Paeoniflorin and glucosides in RPA can reduce the pressure myocardial vascular remodeling and even myocardial remodeling [13]. In addition, paeoniflorin could reverse hypotensive Wistar rats induced by guanethidine, revealing a two-way method of regulating the effects of BP [14].

In our previous study, we evaluated the potential of paeoniflorin enriched extract from Radix Paeoniae Alba (PG) as an antihypertensive agent in spontaneous hypertensive rats (SHR) [15, 16]. We found that PG could decrease blood pressure (SBP, DBP, and MBP) in hypertensive rats, and the mechanism of action was close to its liver protection activity and improvement of endothelial function by reducing ET-1 and increasing NO concentrations. Our survey of literature confirmed that no studies of PG had been conducted to explore BPV and target organ damage in SHR. In our study, we further explored the effect of PG on long- and short-term variability by administering PG at one time or over a long duration to verify the positive effect on BPV. As BPV could cause severe TOD, histopathologic observation of brain, kidney, heart, and thoracic aorta was also performed.

## 2. Animal and Materials

### 2.1. Animal

Male spontaneously hypertensive rats (SHR) (forty-eight weeks old) were all purchased from Beijing Vital River Laboratory Animal Technology Co., Ltd., and the licenses numbers of experimental animal were SCXK (Jing) 2012-0001. All procedures were performed according to protocols following the guidelines for the Use and Care of Laboratory Animals published by the Zhejiang province (2009).

### 2.2. Materials and Reagents

**Figure 1:**
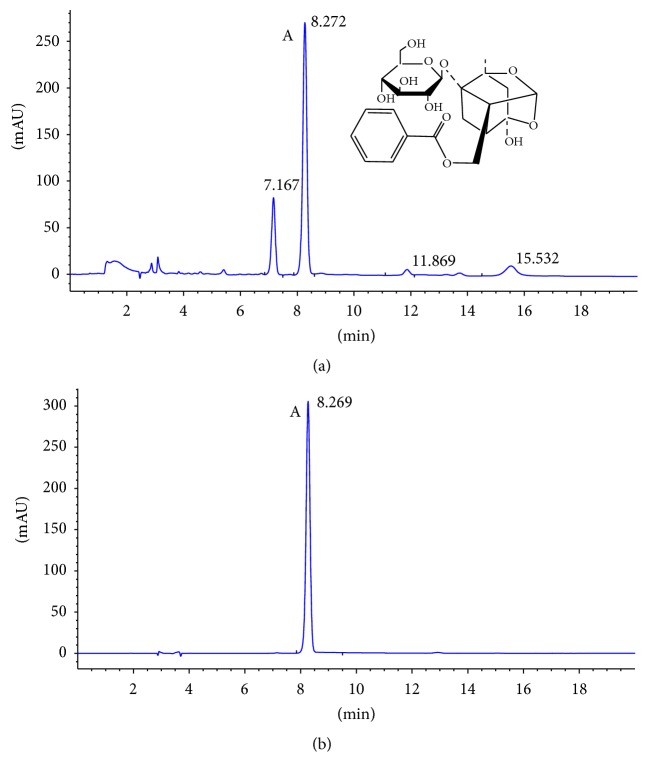
HPLC-DAD analysis of paeoniflorin. (a) HPLC chromatogram of paeoniflorin enriched extract (PG). (b) HPLC chromatogram of standard substance of paeoniflorin. “Peak A” was identified to be paeoniflorin.

Paeoniflorin enriched extract from Radix Paeoniae Alba (PG) was obtained from Zelang Medical Technology Co. (Nanjing, Jiangsu, China) and the purity was 53% detected by HPLC analysis (Figure 1). Metoprolol Tartrate Tablets were purchased from AstraZeneca Pharmaceutical Co., Ltd. (Lot number 1304136). Urea nitrogen (BUN, Lot number 20140617), creatine (Cr, Lot number 20140627), and uric acid (UA, Lot number 20140523) were obtained from Mei Kang Chemical Co. (Ningbo, Zhejiang, China). Hematoxylin (Lot number 20140919), eosin (Lot number 20140919), and Masson's trichrome kit (Lot number 20140919) were purchased from Nanjing Technology Co., Ltd. (Jiangsu, China). Antibodies against endothelial nitric oxide synthase (eNOS, D9A5L) and cyclooxygenase 2 (COX-2 and D5H5) were from Cell Signaling Technology (Beverly, MA). Mouse and Rabbit Specific HRP/DAB (ABC) Detection IHC kit (ab64264) was from Abcam (Cambridge, USA).

### 2.3. HPLC Analysis the Purity of Paeoniflorin

Paeoniflorin enriched extract from Radix Paeoniae Alba (PG) was analyzed with HPLC-DAD. The analysis could simply describe that PG sample was diluted with methanol and filtered through a 0.22 *μ*m membrane filter before put into the system. The Agilent HPLC1200 (Agilent Technologies Inc., Palo Alto, American) was used to determinate the content of paeoniflorin in extracts by C18 column (250 mm × 4.5 mm). The mobile phases were composed of acetic acid and phosphoric acid solution (19 : 81, *V*/*V*), and solvent flow rate was 1 ml/min at column temperature of 25°C. The injection volume was 5 *μ*l. The photodiode array detector was set at 230 nm with a total runtime of 20 min. The HPLC chromatogram of extracts was showed in Figure 1(a).

### 2.4. Implantable Telemetry Technology to Monitor BPV in SHR

Postoperative recovery for one week, selecting three successful surgical SHR, randomly was used for the experiment, based on 24 h dynamic blood pressure (data were not shown). Rats were sequentially given water (model group, MG), metoprolol (20 mg/kg, MP), and PG (90 mg/kg, PG-H; 30 mg/kg, PG-L) every 24 hours according to body weight.

After administration, implantable telemetry technology (Data Sciences International, DSI) collected 24-hour blood pressure (BP) immediately, including systolic blood pressure (SBP), diastolic blood pressure (DBP), and mean arterial blood pressure (MBP). Long-term BPV (SBPV, MBPV, and DBPV) was defined as dividing 24-hour BP into 48 sections, each section for 30 min, and calculated the standard deviation of 48 average values, and the short-term BPV (SBPV, MBPV, and DBPV) was defined as the average values of the 48 sections. The long- and short-term heart variability (HRV) were calculated with the same way the long- and short -term BPV, respectively.

**Figure 2:**
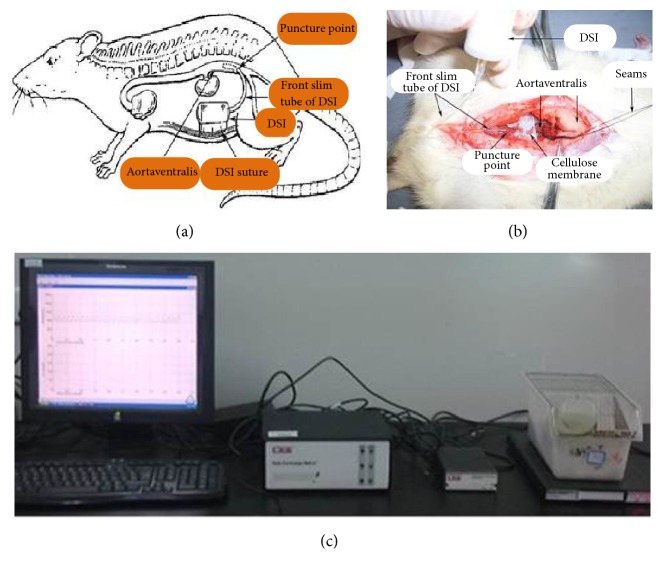
Implantable telemetry technology to monitor dynamic blood pressure in SHR. (a) and (b) The sketch map of intraperitoneal implant site. (c) The transmitting signal device.

The operation was carried out following the procedures presented in literature [17, 18]. Firstly rats were anaesthetized with 3% pentobarbital sodium; then abdominal cavity was exposed and abdominal aorta was separated. The second step was to implant C50-PXT device in the intraperitoneal location. The last step was to suture wound and connect the signal to data quest software in computer to monitor BP for 24 hours. During the surgery and recovery, rats were injected intraperitoneally every day to prevent infection with 0.5 ml penicillin (160 thousand U/ml) and rats could take food and water freely. An illustration of intraperitoneal implant site and transmitting signal device was presented in Figure 2.

### 2.5. Conscious and Freely Moving Animals Dynamic Blood Pressure Analysis System to Monitor BPV in SHR

SHR were randomly assigned to four groups of eight rats each following as model group (MG), metoprolol positive group (20 mg/kg, MP), PG high dose group (90 mg/kg, PG-H), and PG low dose group (30 mg/kg, PG-L), based on SBP (data were not shown). Model group was given water, while others were given corresponding drugs according to body weight daily for seven weeks.

With postoperative recovery for 24 hours, level-headed sober animals dynamic blood pressure analysis system (Shanghai Alcott Biotech Co. Ltd., China) recorded 24-hour BP, including SBP, DBP, and MBP. The computing method of long- and short-term SBPV, MBPV, DBPV, and HRV was the same to former part.

**Figure 3:**
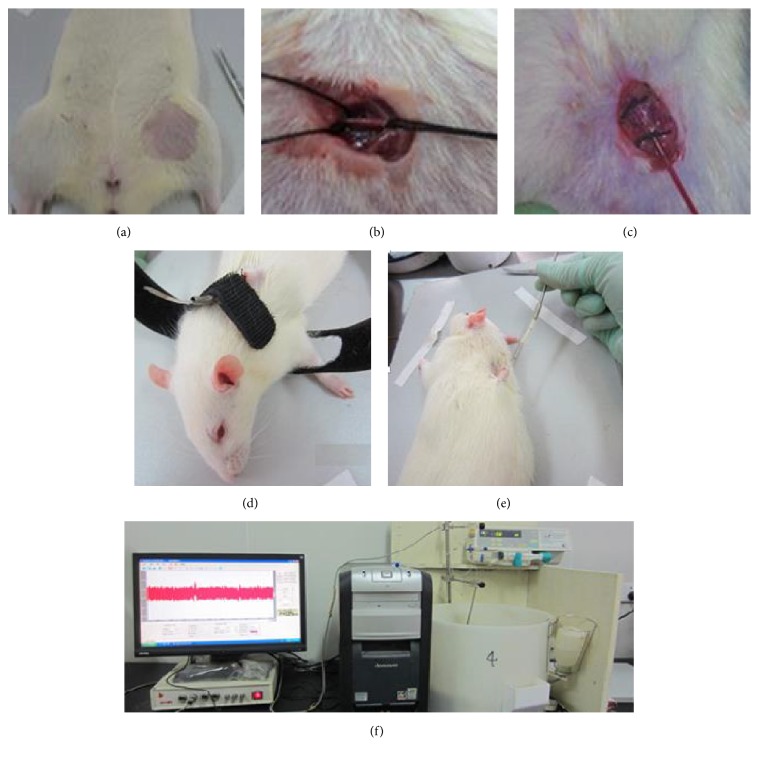
Conscious and freely moving animals dynamic blood pressure analysis system to monitor dynamic blood pressure in SHR. (a)–(e) The sketch map of surgical procedure. (f) The transmitting signal device.

The operation was carried out following the procedure presented in literature [19]. Firstly, rats were anaesthetized with 3% pentobarbital sodium; then the femoral artery was separated and cannula was inserted with the length of weight body × 1% + 1 cm, about 5 cm. Secondly, subcutaneous needle would fix the catheter. At last, level-headed sober animals dynamic blood pressure analysis system was connected and used to monitor BP for 24 hours. During the surgery and recovery, rats were injected intraperitoneally every day to prevent infection with 0.5 ml penicillin (160 thousand U/ml) and rats could take food and water freely. These procedures were displayed in Figure 3.

### 2.6. Renal Function Detected with Biochemical Indexes

After administration for six weeks, SHR were fasted overnight; then 1.25 ml blood was obtained from ophthalmic venous plexus. Blood were water-bathed for 2 hours at 37°C and centrifuged at 3500 rpm for 10 min. The serum was separated to detect the biochemical indexes of BUN, Cr, and UA by TBA-40FR automatic clinical chemistry analyzer (Toshiba, Japan).

### 2.7. Histopathology and Immunohistochemistry Observation of Heart, Brain, Kidney, and Aorta

Meanwhile, aorta, heart, kidney, and brain were put into 4% formalin for fixation. Then, the organs were cut into applicable slice and went through washing and dehydration and embedded to make of tissue wax chunks (MEIKO EC360 Tissue Embedder, Germany). All the specimens were cut into 4 *μ*m thickness (LEICARM2245 slicing machine, Germany) and stained by hematoxylin-and-eosin (H&E). In addition, the specimens of aorta, heart, and kidney were also used for Masson's trichrome staining. For immunohistochemistry (IHC), Mouse and Rabbit Specific HRP/DAB (ABC) Detection IHC kit was used for the development of reaction of eNOS in aorta, as well as COX-2 in heart and kidney, and tissues were counterstained with hematoxylin. Histopathology observation was used with biological microscope B5-223IEP (Germany).

### 2.8. Statistical Analysis

All data were expressed as the mean ± standard deviation and subjected to *t*-test. When compared with the model group before treatment, the data were subjected to paired-sample *t*-tests. When compared with the same period of the model group, the data were subjected to independent-sample *t*-tests. *P* value of <0.05 was considered statistically significant. All analyses were performed by using an updated version of SPSS 15.0 software.

## 3. Results

### 3.1. Paeoniflorin Enriched Extract Has Slight Effect on 24-Hour Total Blood Pressure of SBP after Single Administration

**Figure 4:**
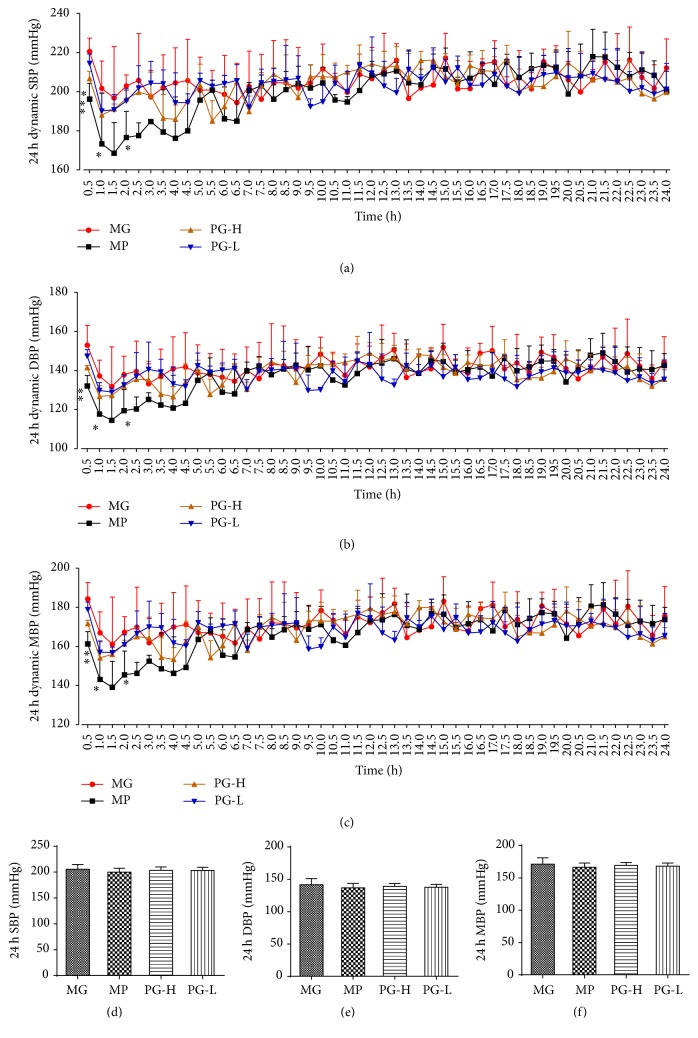
Effect on 24-hour total blood pressure of SBP, DBP, and MBP after being intervened by paeoniflorin enriched extract (PG) at one time. (a) The 24-hour dynamic SBP. (b) The 24-hour dynamic DBP. (c) The 24-hour dynamic MBP. (d) Analysis of 24-hour total SBP. (e) Analysis of 24-hour total DBP. (f) Analysis of 24-hour total MBP. SBP: systolic blood pressure; DBP: diastolic blood pressure; MBP: mean blood pressure; MG: model group before treatment; MP: metoprolol positive group; PG-H: PG high dose group (90 mg/kg); PG-L: PG low dose group (30 mg/kg). Data were mean ± SD (*n* = 3). ^*∗*^*P* < 0.05 versus MG; ^*∗∗*^*P* < 0.01 versus MG.

First, we evaluated the effect of 24-hour dynamic BP in SHR with single dose of metoprolol and paeoniflorin enriched extract (PG). As a result, metoprolol 20 mg/kg could decrease SBP, DBP, and MBP at the time of 0.5, 1, and 2 h, compared with the model group before treatment (*P* < 0.05 and 0.01), and PG 90 mg/kg exhibited significant influence on SBP at the time of 0.5 h (*P* < 0.05) (Figures 4(a)~4(c)). In contrast, PG 30 mg/kg had no statistic difference on SBP, DBP, and MBP at any time. Meanwhile, the data of 24-hour total SBP, DBP, and MBP showed that PG and metoprolol, administration for just once, had no significantly effect on those parameters (Figures 4(d)~4(f)).

### 3.2. Paeoniflorin Enriched Extract Does Not Aggravate Long- and Short-Term Blood Pressure Variability of SBPV, DBPV, MBPV, and HRV after Single Administration

**Figure 5:**
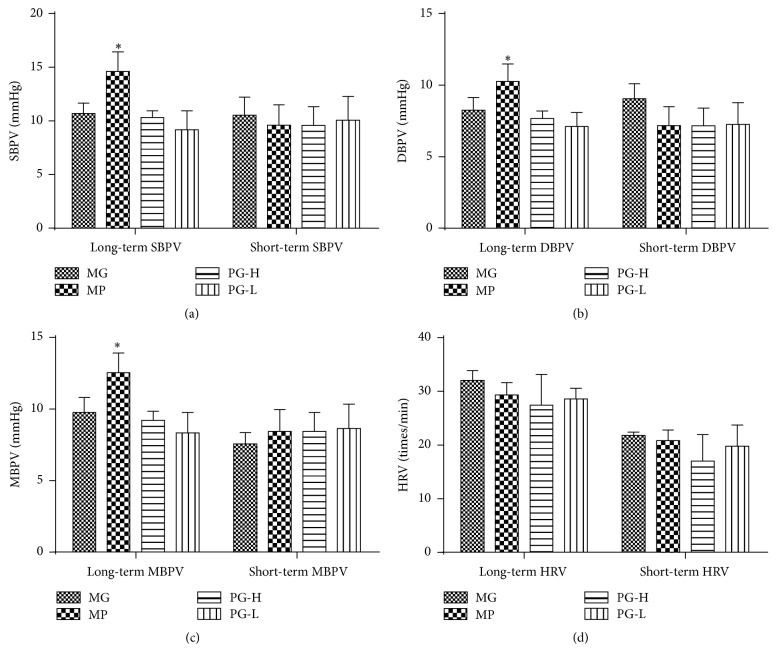
Effect on long- and short-term blood pressure variability of SBPV, DBPV, MBPV, and HRV after being intervened by paeoniflorin enriched extract (PG) at one time. (a) Analysis long- and short-term SBPV. (b) Analysis long- and short-term MBPV. (c) Analysis long- and short-term DBPV. (d) Analysis long- and short-term HRV. SBPV: systolic blood pressure variability; DBPV: diastolic blood pressure variability; MBPV: mean blood pressure variability; HRV: heart rate variability; MG: model group before treatment; MP: metoprolol positive group; PG-H: PG high dose group (90 mg/kg); PG-L: PG low dose group (30 mg/kg). Data were mean ± SD (*n* = 3). ^*∗*^*P* < 0.05 versus MG.

After single dose, when compared with the model group before treatment, metoprolol 20 mg/kg could notably increase SBPV, DBPV, and MBPV (*P* < 0.05), while PG had no visible influence on long- and short-term of SBPV, DBPV, MBPV, and HRV (Figure 5).

### 3.3. Paeoniflorin Enriched Extract Ameliorates 24-Hour Total Blood Pressure of SBP, DBP, and MBP after Administration for Seven Weeks

**Figure 6:**
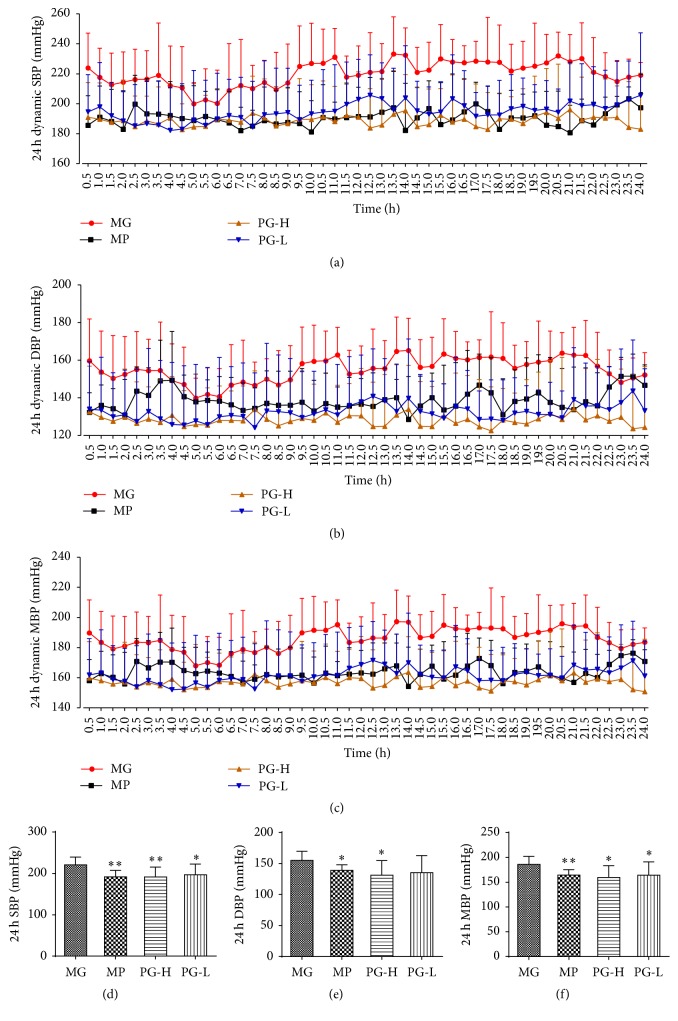
Effect on 24-hour total blood pressure of SBP, DBP, and MBP after being intervened by paeoniflorin enriched extract (PG) for seven weeks. (a) The 24-hour dynamic SBP. (b) The 24-hour dynamic DBP. (c) The 24-hour dynamic MBP. (d) Analysis of 24-hour total SBP. (e) Analysis of 24-hour total DBP. (f) Analysis of 24-hour total MBP. SBP: systolic blood pressure; DBP: diastolic blood pressure; MBP: mean blood pressure; MG: model group; MP: metoprolol positive group; PG-H: PG high dose group (90 mg/kg); PG-L: PG low dose group (30 mg/kg). Data were mean ± SD (*n* = 8). ^*∗*^*P* < 0.05 versus MG; ^*∗∗*^*P* < 0.05 versus MG.

From the former part experiment, PG had slight effect on blood pressure for single administration; then the effect of long-time administration performed further. The results of 24-hour dynamic BP suggested that PG and metoprolol all significantly amended the BP at monitoring time, while PG (30 and 90 mg/kg) were provided with the high stability of BP obviously (Figures 6(a)~6(c)). The data of 24-hour total SBP, DBP, and MBP showed that PG 90 mg/kg could significantly lower those parameters (*P* < 0.05, 0.01), and PG 30 mg/kg could also remarkably decrease 24-hour total SBP and MBP (*P* < 0.05), compared with the model group (Figures 6(d)~6(f)).

### 3.4. Paeoniflorin Enriched Extract Decreases Long- and Short-Term Blood Pressure Variability of SBPV, DBPV, and MBPV after Administrating for Seven Weeks

**Figure 7:**
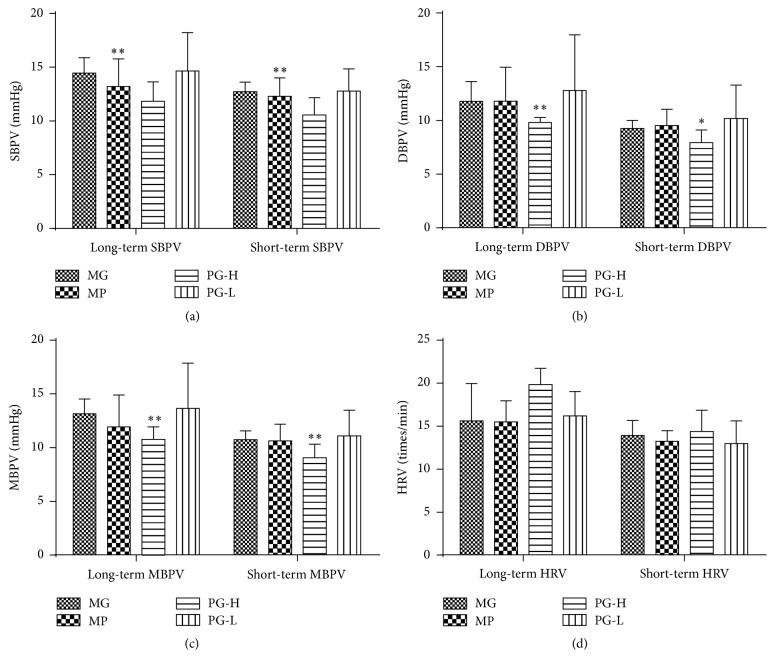
Effect on long- and short-term blood pressure variability of SBPV, DBPV, MBPV, and HRV after being intervened by paeoniflorin enriched extract (PG) for seven weeks. (a) Analysis long- and short-term SBPV. (b) Analysis long- and short-term MBPV. (c) Analysis long- and short-term DBPV. (d) Analysis long- and short-term HRV. SBPV: systolic blood pressure variability; DBPV: diastolic blood pressure variability; MBPV: mean blood pressure variability; HRV: heart rate variability; MG: model group; MP: metoprolol positive group; PG-H: PG high dose group (90 mg/kg); PG-L: PG low dose group (30 mg/kg). Data were mean ± SD (*n* = 8). ^*∗*^*P* < 0.05 versus MG; ^*∗∗*^*P* < 0.05 versus MG.

Compared with model group, PG 90 mg/kg significantly improved the long-term blood pressure variability of SBPV, DBPV, and MBPV (*P* < 0.01), as well as the short-term blood pressure variability of SBPV, DBPV, and MBPV (*P* < 0.01, 0.05) (Figures 7(a)~7(c)). And PG 30 mg/kg and metoprolol 20 mg/kg had no visible difference on those parameters (Figure 7).

### 3.5. Protective Effect on Aorta Pathological Changes after Administration with Paeoniflorin Enriched Extract for Seven Weeks

**Figure 8:**
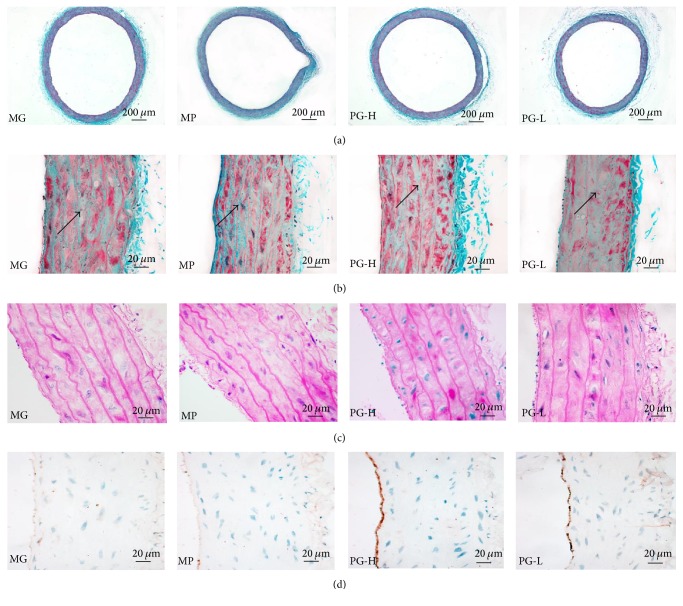
Effect on vascular lesions after being intervened by paeoniflorin enriched extract (PG) for seven weeks. (a) Representative photomicrograph of histopathologic observation for aorta by Masson's trichrome staining (×40). (b) Representative photomicrograph of histopathologic observation for aorta by Masson's trichrome staining (×400). (c) Representative photomicrograph of histopathologic observation for aorta by hematoxylin-and-eosin staining (H&E) (×400). (d) Representative photomicrograph of the eNOS expression in aorta by immunohistochemistry (IHC) (×400). MG: model group; MP: metoprolol positive group; PG-H: PG high dose group (90 mg/kg); PG-L: PG low dose group (30 mg/kg).

Masson's trichrome staining was used to estimate overall collagen deposition in the aorta as indicated by the density of blue staining (Figures 8(a) and 8(b)). Collagen deposition significantly increased in the model group, while PG mitigated it in a dose-dependent manner. In addition, we also examined pathological changes of the aorta with H&E staining as manifested by the aorta endothelial shedding, the thickness of the media increase, and vascular smooth muscle cells (VSMC) hypertrophy in the model group (Figure 8(c)). To confirm the aorta endothelial lesions furtherly, then we performed the expression of eNOS in the aorta endothelium with IHC, which revealed that the eNOS expression in the aorta endothelium decreased in the model group (Figure 8(d)). And PG could reverse those lesions. In common, those data provided favorable evidence that PG could alleviate the hypertension induced histopathology injury of aorta.

### 3.6. Protective Effect on Heart Pathological Changes after Administration with Paeoniflorin Enriched Extract for Seven Weeks

**Figure 9:**
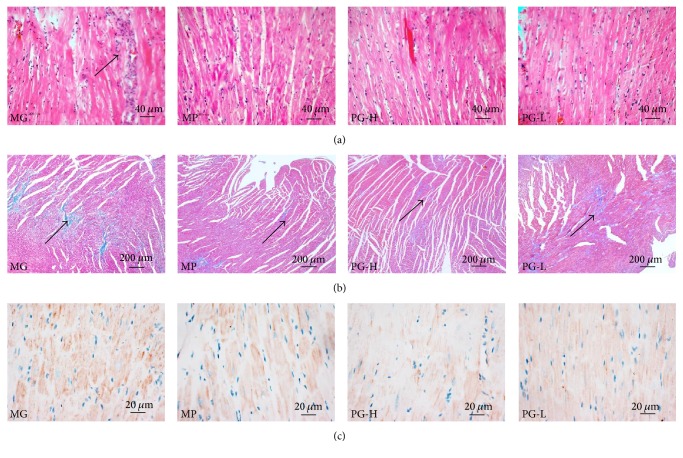
Effect on histopathology of heart after being intervened by paeoniflorin enriched extract (PG) for seven weeks. (a) Representative photomicrograph of histopathologic observation for heart by hematoxylin-and-eosin staining (H&E) (×200). (b) Representative photomicrograph of histopathologic observation for heart by Masson's trichrome staining (×40). (c) Representative photomicrograph of the COX-2 expression in heart by immunohistochemistry (IHC) (×400). MG: model group; MP: metoprolol positive group; PG-H: PG high dose group (90 mg/kg); PG-L: PG low dose group (30 mg/kg).

As shown in the heart H&E and Masson's trichrome staining, the cardiac muscle cells were wider and the nucleus was larger occasionally accompanied by inflammatory cell infiltration and there were lots of collagen deposition in the model group (Figures 9(a) and 9(b)). To further demonstrate inflammatory lesions, we examined the expression of COX-2 in heart with IHC. The results showed that COX-2 was highly expressed in the model group (Figure 9(c)). In contrast, PG could markedly alleviate those symptoms, which hinted that PG protected SHR against hypertension induced cardiac injury.

### 3.7. Protective Effect on Kidney Pathological Changes and Function after Administration with Paeoniflorin Enriched Extract

**Figure 10:**
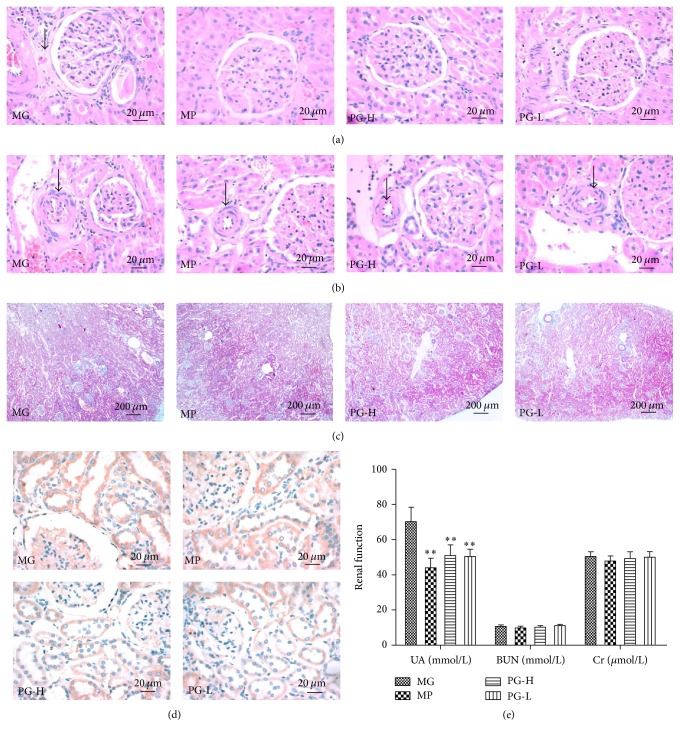
Effect on histopathology of kidney and renal function after being intervened by paeoniflorin enriched extract (PG). (a) Representative photomicrograph of histopathologic observation for glomerulus by hematoxylin-and-eosin staining (H&E) (×400). (b) Representative photomicrograph of histopathologic observation for glomerular afferent arteries by H&E (×400). (c) Representative photomicrograph of histopathologic observation for kidney by Masson's trichrome staining (×40). (d) Representative photomicrograph of the COX-2 expression in kidney by immunohistochemistry (IHC) (×400). (e) The renal function indexes of serum UA, BUN, and Cr. MG: model group; MP: metoprolol positive group; PG-H: PG high dose group (90 mg/kg); PG-L: PG low dose group (30 mg/kg). Data were mean ± SD (*n* = 8). ^*∗∗*^*P* < 0.01 versus MG.

In this part, we defined the impact of PG on kidney injury by biochemical analysis of the serum BUN, UA, and Cr levels in SHR. Compared with model group, PG 90 mg/kg and 30 mg/kg had an significant effect on decreasing the level of UA (*P* < 0.01) (Figure 10(e)). However, PG had no significant effect on serum BUN and Cr. Then, we performed histological analysis with H&E and Masson's trichrome staining of renal sections. H&E staining revealed the presence of glomerular wall thickening (Figure 10(a)) and luminal stenosis in the arterioles (Figure 10(b)). Moreover obvious collagen deposition in glomerular wall but no intertubular fibrosis was noted in model group as manifested by the Masson's trichrome staining (Figure 10(c)). And with highly expressed in the model group, the expression of COX-2 in kidney was similar in heart (Figure 10(d)). Of note, PG significantly attenuated these pathological changes. Collectively, our data provided convincing evidence that PG protected SHR against hypertension induced kidney injury.

### 3.8. Protective Effect on Brain Pathological Changes after Administration with Paeoniflorin Enriched Extract for Seven Weeks

**Figure 11:**
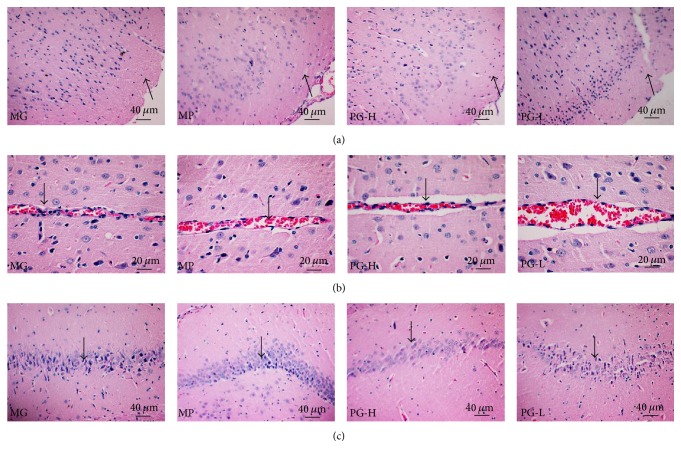
Effect on histopathology of brain after being intervened by paeoniflorin enriched extract (PG) for seven weeks. (a) Representative photomicrograph of histopathologic observation for cerebral cortex by hematoxylin-and-eosin staining (H&E) (×200). (b) Representative photomicrograph of histopathologic observation for cerebral cortex blood vessel by H&E (×400). (c) Representative photomicrograph of histopathologic observation for hippocampal CA1 area by H&E (×200). MG: model group; MP: metoprolol positive group; PG-H: PG high dose group (90 mg/kg); PG-L: PG low dose group (30 mg/kg).

In this section, we examined the protective effect of PG on brain injury with H&E staining. In the model control group, the cortical cells arranged in disorder and decreased (Figure 11(a)). The cortical vascular endothelial cell was swelling (Figure 11(b)). Meanwhile, by observing the hippocampal CA1 area, the pyramidal cell layer became thinner, less, and disordered, and the neurons were degenerated and necrosed obviously (Figure 11(c)). Compared with the model control group, the PG (90 mg/kg) in different degrees improve the cortical cells lesions, alleviate the swelling of cortical vascular endothelial cells, and attenuate the pyramidal cell layer in hippocampal CA1 area. Although we only used H&E staining to observe brain lesions, severe brain injury could be clearly observed in the model control group and PG had significant protective effect on the brain injury in SHR.

## 4. Discussion

**Figure 12:**
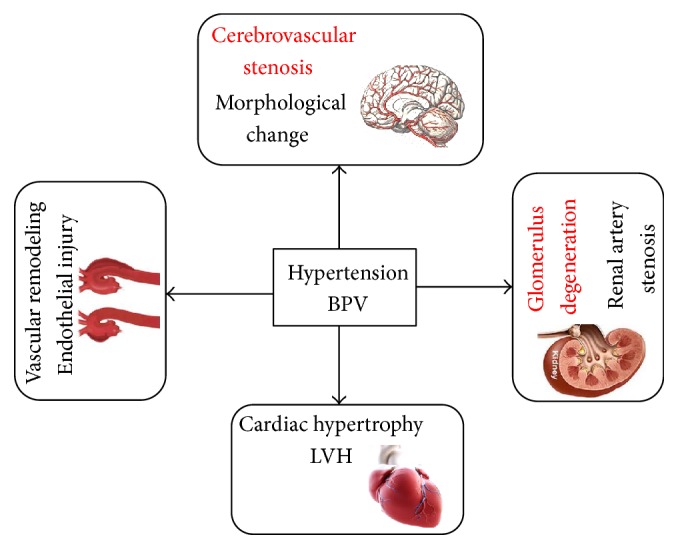
Hypertensive target organ damage (TOD) in different tissues.

Hypertension is a disease characterized by high arterial pressure. Sustained high blood pressure leads to cerebral embolism, cardiac failure, renal failure, and other complications. TOD is caused not only by hypertension but also by BPV, which is independent of low mean systolic blood pressure [4, 20]. Therefore, the treatment of hypertension should focus not only on the effective control of blood pressure, but also on the protection of target organs to reduce complications [6]. Many researchers confirmed that BPV could cause TOD independently, even within normal blood pressure [21, 22]. There is emanating evidence that BPV is an independent predictor of hypertensive TOD and cardiovascular events [23]. An illustration of TOD is presented in Figure 12.

In the early stage of our research, PG exhibited a definite antihypertensive effect on SHR through liver protection activity and improvement of endothelial function by regulating serum NO and endothelin (ET) levels. However, the effect of PG on blood pressure fluctuation has not been evaluated. On the basis of the previous research, single-dose and long-term administration of PG were conducted to investigate its effect on BPV in SHR in this study. The experimental results showed that single-dose administration of PG, unlike metoprolol, significantly reduced blood pressure in rats initially, without aggravating long- and short-term BPV. Long-term administration of PG could not only significantly reduce the 24 h blood pressure but also decrease BPV (SBPV, DBPV, and MBPV). By contrast, metoprolol significantly reduced the 24 h blood pressure; however the trend fluctuated, showing no significant effect on SBPV, DBPV, and MBPV. This effect might be the advantage of using PG as an antihypertensive. The effect of *β*-blockers on blood pressure fluctuation remains inconclusive. Beta-blockers might increase in enhanced BPV [20], which may be attributed to nonselective *β*-blockers; high selectivity in *β*-blockers exerts no such effect [24].

Vascular cell proliferation, apoptosis, inflammation, fibrosis, and other complex processes change the vascular structure in patients with BPV [25]. The SHR is a stable model for examining the development and complications of hypertension. Increased collagen deposition, endothelial cell abnormalities, and abnormal aortic wall cell proliferation of the media were observed in the 42-week-old SHR [26]. The SHR exhibited a significant reduction of endothelial nitric oxide synthase (eNOS) protein expression in the aortic endothelium [27]. An earlier study suggested that paeoniflorin could promote blood vessel wall function by releasing the relaxation factor of NO on isolated thoracic aorta rings of SD rats [12]. Likewise, our previous research proved that PG could upregulate serum NO in SHR [15]. Endothelial NOS is the main limiting factor of NO generation, forcefully causes vasodilation, and inhibits the proliferation of vascular smooth muscle cells [28]. The findings in the current study suggested that PG could improve endothelial shedding, relieve hypertrophy of smooth muscle cells, improve collagen fiber hyperplasia, and increase eNOS expression in the aorta, which hinted that PG could alleviate the hypertension induced histopathology injury of aorta.

Enhanced BPV could trigger a change in pathological cardiac hypertrophy via mechanical stress fluctuation in the cardiomyocytes [29]. That is to say, increased BPV may be responsible for the pathogenesis of hypertrophic cardiac response [30]. The clinical test showed that an increase in BPV over 24-hour evaluation period with ambulatory blood pressure monitoring was linked to a higher degree of hypertensive cardiovascular complications [31, 32]. Studies indicated that BPV was highly correlated with cardiovascular complications, and short-term variability could predict the close links to BPV and early left ventricular systolic dysfunction [33, 34]. BPV also changes in the myocardial structure [35]. SHR aged 56 weeks developed end-stage hypertensive heart disease, enlargement of cardiomyocytes enlargement, and fibrosis [36], which were also were found in SHR aged 20 weeks [37]. Moreover, the expression of COX-2 in cardiomyocytes was significantly correlated with their size [38], and COX-2 might be one of the important indicators of systemic inflammation [39, 40]. Paeoniflorin has been reported to prevent upregulations of proinflammatory mediator COX-2 in ischemia-induced brain damage and rheumatoid arthritis rats [41, 42]. The results obtained in this study revealed that PG could improve myocardial inflammation, ameliorate collagen deposition, and decrease COX-2 expression in heart.

The kidneys target organs that not only are prone to hypertension induced injury but also are also involved in exacerbating the development of hypertension. An increase in short-term BPV may be positively correlated with impaired renal function, as determined by microalbuminuria or glomerular filtration rate [27, 28]. Glomeruli, tubules-interstitium, and renal vascular lesions were significantly increased in 12-week-old SHR [43, 44]. Renal COX-2 expression was also increased in hypertension mouse [43]. In this study, histopathologic observation of renal tissues indicated that glomerular arteries led to stenosis, thickening of the glomerular wall capsule, collagen deposition, and increase in COX-2 expression in the SHR model. PG could improve the aforementioned symptoms suggesting that PG exhibited a renal protective effect in SHR.

BPV could also lead to brain damage, including cerebral vascular lesions and histomorphological changes in the brain [45]. Cerebrovascular lesions are mainly manifested as hypertrophic and remodeling lesions. Histomorphological changes mainly occur in the frontal lobe, occipital lobe, and hippocampus. Previous studies have indicated that SHR share behavioral and neuropathological characteristics in 22-week-old SHR [46]. Paeoniflorin could attenuate brain damage in rats and mouse via inflammatory signaling pathways [47, 48]. In this study, PG could variably increase the number of cortical cells, alleviate the swelling of cortical vascular endothelial cells, and attenuate the pyramidal cell layer in the hippocampal CA1 area.

The current animal models, used to study for hypertension, are mainly hereditary hypertensive animal models (spontaneously hypertensive rats (SHR) [49, 50], stroke-prone spontaneously hypertensive rats [51], Dahl salt-sensitive hypertensive rats [52], etc.), renovascular hypertensive animal models (2-kidney-1-clip, 2K1C [53] and 2-kidney-2-clip, 2K2C [54], etc.), drug-induced hypertensive animal models (angiotensin-induced hypertension [55], L-nitro arginine methyl ester induced hypertension [56], etc.), metabolic hypertensive animal models (excessive alcohol intake and high fat diet induced hypertensive rats [16], high-purine diet induced hypertensive rats [57], high-glucose/fat diet induced hypertensive rats [58], etc.), and so on. SHR, hypertensive spontaneous rate of 100%, were nurtured form Wistar rats by Okamoto and Aoki in 1963 [59], which is internationally recognized as the most comparable in characteristics with human essential hypertension (EH). With the development of the disease, SHR present heart [37], brain [46], kidney [43, 44], blood vessels [26], and other types of target organ damage. Classic BPV animal model, with surgery to remove sinus nerve bow (sinoaortic-denervated, SAD), was created successfully by the Krieger in 1964 [60], with the limitations of a high mortality rate and pure neurogenicity [61]. However, the BPV of SHR was positively correlated with it age. The BPV in 40-week-old SHR is higher than that in 16-week-old SHR [62] and the BPV in 7-month-old and 5-month-old SHR is higher than that in 3-month-old SHR [63]. So, we selected the 48-week-old SHR to evaluate the protective effect of PG on BPV and TOD in present research. Continuously monitoring 24-hour blood pressure is the basic guarantee of evaluation BPV. Methods are mainly application with noninvasive telemetry system [64], implantable telemetry technology [17, 18], and conscious and freely moving animals dynamic blood pressure analysis system [19]. Noninvasive telemetry system is mainly used for monitoring 24-hour blood pressure of human and large animals as dogs and monkeys, with a vest and no surgery. Implantable telemetry technology, with the signal transmitter and the pressure signal device embedded in the abdominal cavity, can collect data in conscious and freely moving animals for a long-term, when the animals return to normal after surgery. Using implantable telemetry technology could reduce pain and stress of animals and reduce the number of animals by improving data accuracy and self-control [65]. Conscious and freely moving animals dynamic blood pressure analysis system, with arterial catheterization and continuous heparinization to ensure the transmission of the signal, will produce a certain degree of pain and stress and could not collect data for a long-term [63]. Therefore, to evaluate the protective effect of PG on BPV, implantable telemetry technology (Figure 2) was performed to monitor the 24-hour BPV in 3 SHR by self-control, and conscious and freely moving animals dynamic blood pressure analysis system (Figure 3) were performed to monitor the 24-hour BPV in 32 SHR.

In conclusion, abnormal BPV and TOD of heart, brain, kidney, and aorta were observed in SHR of this study, which is consistent with the other researcher reports. And paeoniflorin enriched extract (PG) could reduce BPV, stabilize blood pressure, and reverse the eNOS or COX-2 expression to mitigate target organ damage (TOD) in SHR. These findings provide convincing evidence that PG, with protective effect on BPV and TOD in SHR, can be used to treat hypertension. However, the mechanisms of the increased BPV resulting in TOD were not elucidated in the present study, which may be related to chronic inflammation [66] and microcirculation [67].
